# Light-responsive biomaterials for ocular drug delivery

**DOI:** 10.1007/s13346-022-01196-5

**Published:** 2022-06-24

**Authors:** Hend A. M. Abdelmohsen, Nikki A. Copeland, John G. Hardy

**Affiliations:** 1 Department of Chemistry, Lancaster University, Lancaster, LA1 4YB Lancashire UK; 2 Division of Biomedical and Life Sciences, Lancaster University, Lancaster, LA1 4YW Lancashire UK; 3 Department of Pharmaceutics and Industrial Pharmacy, Faculty of Pharmacy, Ain Shams University, African Union Organization Street, Abbassia, Cairo, 11566 Egypt; 4 Materials Science Institute, Lancaster University, Lancaster, LA1 4YB Lancashire UK

**Keywords:** Ocular drug delivery, Stimuli-responsive, Light-triggered, Light-responsive, Photochemistry, Photobiology

## Abstract

Light-responsive biomaterials can be used for the delivery of therapeutic drugs and nucleic acids, where the tunable/precise delivery of payload highlights the potential of such biomaterials for treating a variety of conditions. The translucency of eyes and advances of laser technology in ophthalmology make light-responsive delivery of drugs feasible. Importantly, light can be applied in a non-invasive fashion; therefore, light-triggered drug delivery systems have great potential for clinical impact. This review will examine various types of light-responsive polymers and the chemistry that underpins their application as ophthalmic drug delivery systems.

**Graphical abstract:**

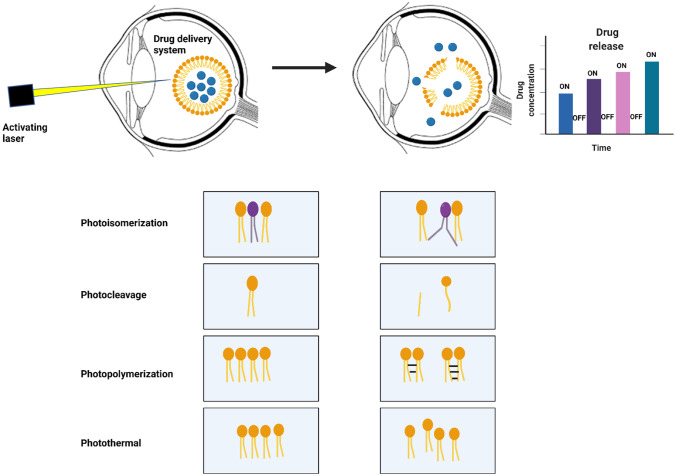

## Introduction

**Fig. 1:**
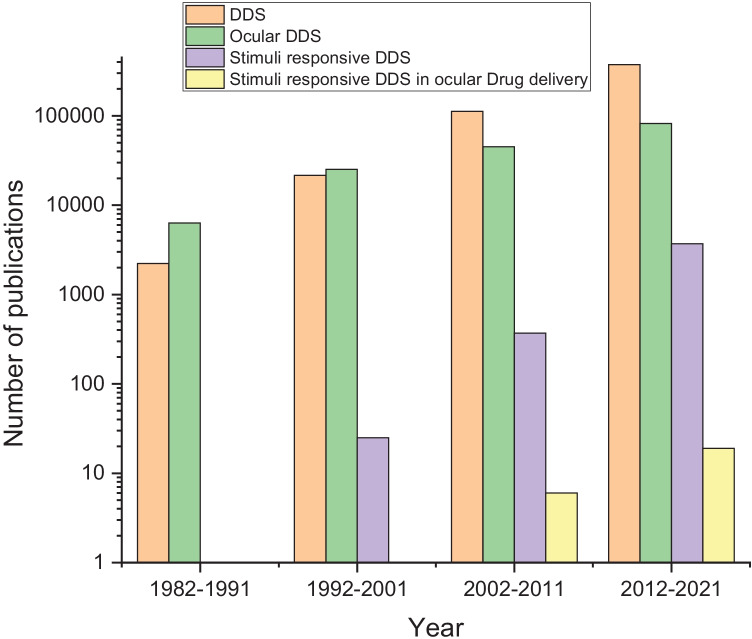
Number of publications on related topics in the Web of Science database with respect to time (search date: 1–3-2022)

Among the various routes of administration for drugs in human and veterinary medicine, ophthalmic drug delivery (Fig. 1) is one of the most challenging despite the simple accessibility of the eyes, and ocular protection by various barriers and defense mechanisms necessitates the development of efficient drug delivery systems (DDSs) [1–4]. The anterior and posterior segment of the eye is affected by several ocular diseases that lead to vision threatening, degeneration of the retina, and blindness. Diseases affecting anterior segment include, but not limited to glaucoma, allergic conjunctivitis, anterior uveitis, and cataract, while age-related macular degeneration, diabetic macular edema, proliferative vitreoretinopathy, posterior uveitis, cytomegalovirus infection, and glaucoma affect the posterior parts of the eye [5–7]. In principle, ocular tissues are accessible to light, and, therefore, light-responsive DDSs [8, 9] may find clinical applications in ophthalmology. Investigation of light delivery systems in ocular drug delivery is a topic of emerging interest in this field, and this review offers a concise coverage of these DDS.

### Barriers to ocular drug delivery

**Fig. 2:**
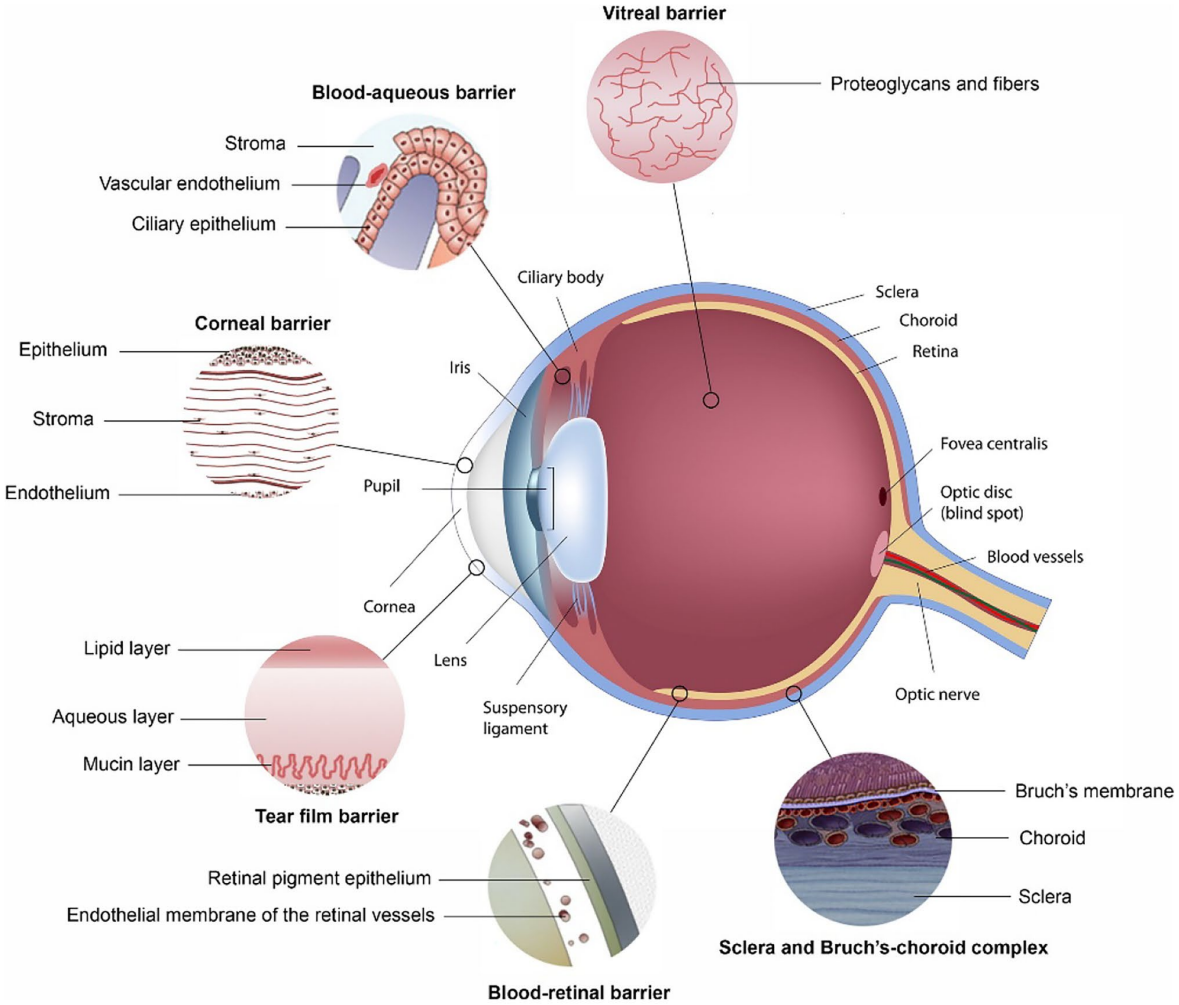
Physiological barriers in ocular drug delivery. Reproduced from [23] with permission

As an organ, the eyes are divided anatomically into the anterior and posterior segments. The anterior segment includes the cornea, aqueous humor, conjunctiva, iris, and lens, while the posterior segment includes the retina–choroid, sclera, optic nerve, and vitreous humor [10, 11]. A combination of factors provides challenges for ocular drug delivery (Fig. 2). The tear film is the first defensive barrier for topical medication (composed of the lipid, aqueous layer, and mucin layer). The rapid turnover rate of tears (0.5–32.2 µL/min), nasolacrimal duct drainage (the main route of tear discharge), and reflex tear production are the main reasons for drug loss [12]. The cornea also restricts the entry of foreign substances into the eyes; corneal layers include epithelium, Bowman’s layer, stroma, Descemet’s membrane, and endothelium, each layer of which has a different polarity, and the corneal epithelium and stroma are considered to be the main obstacles [13]. The tight junctions and hydrophobicity of the corneal epithelium could block the diffusion of hydrophilic drugs [14]. The corneal stroma accounts for 90% of the corneal thickness, which provides a hydrophilic barrier to molecular transport for hydrophobic drugs. The corneal endothelium is leaky and has no significant effect on molecules penetration that is beneficial to the exchange of nutrients between the corneal stroma and aqueous humor. Importantly, the use of nanomedicine can effectively enhance the corneal permeability of the drug that was reviewed in details in [15–17]. The conjunctiva is a mucosa comprised of 2–3 layers of epithelial cells and vascularized connective tissue. Moreover, the conjunctival epithelium is also a rate-limiting step for hydrophilic drugs diffusion and the non-corneal pathway [18]. In comparison with the cornea, the conjunctiva facilitates the absorption of large and hydrophilic drugs like small interfering RNA and peptides; however, its vasculature may aid in drugs clearance to the systemic circulation that would reduce the drug bioavailability [19]. The blood-eye barrier is one of the main barriers in the eyes, which is comprised of the blood-aqueous barrier (BAB) and the blood-retinal barrier (BRB). The BAB is composed of iris epithelial cells, capillary endothelial cells, and non-pigmented epithelium of the ciliary body that all contain tight junctions. The tight junctions hinder the drug transport from the plasma to aqueous humor after systemic administration [18]. The BAB could limit the drug bioavailability in aqueous humor by its elimination via the uveoscleral pathway together with counterflow of the aqueous humor [20]. In conclusion, the tear film, the cornea, and the BAB are the main obstacles in the anterior eye chamber. In addition, the transparent aqueous humor and the lens located behind the cornea also hinder the drug from reaching the posterior eyes [18, 21].

In the posterior eye compartment, the sclera, choroid, vitreous body, and BRB constitute the main barriers. The sclera occupies the largest surface area of the eyeball (typically > 80%) [22], with a slightly weaker barrier effect in comparison with cornea; it is mainly composed of collagen fibers, and the permeability of the sclera is related to the molecular weight and charge of the drug [13], where low molecular weight drugs that are negatively charged tend to penetrate the sclera effectively [18]. The choroid has a rich vascular network, where high blood perfusion can facilitate drug elimination from the eye [23]. The vitreous body is mainly constituted of different types of collagen fibers and hyaluronic acid [23], and the diffusion rate of drugs in the vitreous body is controlled by their structure and surface charge, where the diffusion rates of the molecules are inversely proportional to their molecular weight; furthermore, the permeation of positively charged molecules is retarded by their electrostatic interaction with the anionic carboxylates displayed on hyaluronic acid, potentially leading to aggregation and immobilization [19]. The inner limiting membrane is located between the vitreous body and the retina, and it is the main barrier for intravitreal drug transport to the retina [20]. Finally, the BRB is a physiological barrier divided into internal BRB that is formed by tight junctions between retinal capillary endothelial cells. The external BRB is formed by tight junctions between retinal pigment epithelial cells, it regulates the influx of ions, proteins, and water; the tight junctions between the endothelial cells and efflux transporters can hinder the diffusion of positively charged hydrophilic therapeutic agents from plasma to the retina [24]; and passive diffusion through the BRB can quickly eliminate lipophilic molecules that enter the vitreous body [24, 25].

### Ocular drug delivery routes

**Fig. 3:**
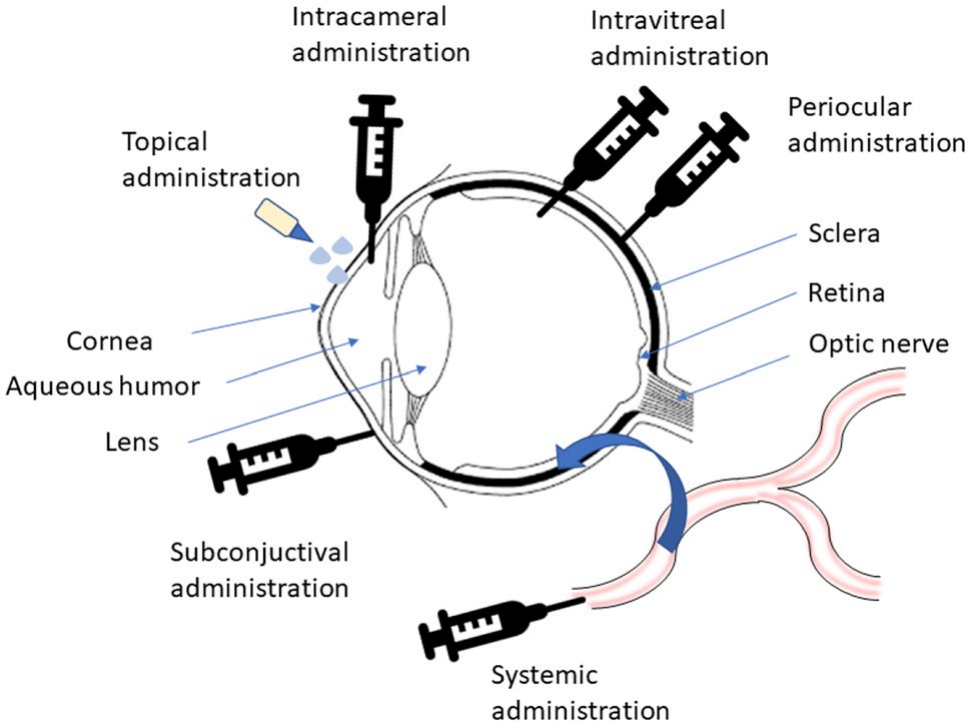
Anatomical structure of the eye with possible routes for drug administration

Drug delivery to the anterior and posterior segments of the eye can be achieved by various ways, including topical, systemic, and ocular injection routes (Fig. 3). In topical administration, although commonly implemented therapies like drops of drug solutions/suspensions are easy to manufacture with high patient compliance, their application suffers from many limitations due to the anatomical, physiological, and biochemical features of the eye that result in poor bioavailability, mainly attributed to rapid tear clearance, small eye capacity, and blinking which reduce drug residence time, concentration, and availability for absorption [26–30]. Dosage forms including viscous ointments and gels have been formulated to overcome these issues and enhance ocular retention time (noting that patient compliance can be diminished if the DDSs result in blurred vision) [31]. Systemic administration has been used for ocular delivery; however, typically only 1–2% of the administered dose reaches the target site. This could be attributed to the eye which has a low blood supply compared with the whole body, and the tight junctions of the retinal pigment epithelial cells hinder drug diffusion to the retina; a frequent administration would be necessary to achieve satisfactory ocular concentrations thus leading to systemic side effects [19]. On the other hand, ocular injections could be delivered through intracameral, subconjunctival, periocular, and intravitreal routes. Intracameral administration is an approach of injecting drugs directly into the anterior segment of the eye. As it avoids the corneal barrier, it can provide higher drug concentrations in the aqueous humor in comparison to eye drops. Subconjunctival injections can be administered under the eyeball conjunctiva (epibulbar) or underneath the conjunctiva lining the eyelid (subpalpebral). It allows drugs to bypass the corneal and conjunctival barriers with less trauma. However, the major drawback of these routes is the shorter retention time [32]. Drug delivery via the periocular route, which includes subconjunctival, subtenon, peribulbar, posterior juxtascleral, and retrobulbar injections, may also be effective in delivering drugs to the posterior eye segment. In comparison with intravitreal administration, periocular administration is relatively less invasive and can reduce complications associated to intravitreal administration [33]. However, drug diffusion is hindered by the episclera, sclera, choroid, and Bruch’s membrane before reaching the retina, and clearance from the administration site could result in significant drug loss [34]. Intravitreal administration involves intravitreal injections and intravitreal implants. The intravitreal injections are able to achieve therapeutic drug concentrations in the posterior eye compartment where drug solutions are directly injected into the vitreous body as it overcomes the majority of elimination mechanisms and barriers. Attempts to treat diseases in the posterior segment of the eyes usually need frequent intravitreal injections every 1 to 2 months. Nevertheless, drug delivery to the posterior segment of the eye through intravitreal injections has a high risk of complications, such as retinal detachment, inflammation (e.g., iritis/uveitis), infection, and vitreous hemorrhage [35]. Intravitreal implants are pharmaceutically designed systems to deliver drugs for prolonged periods (1–6 months) alleviating the requirement of multiple injections and developing inflammatory processes and infections associated with intravitreal injections [36]. These systems are also obtainable in two different chemical structures–the biodegradable and the non-biodegradable ones and usually requires one or two surgical procedure for administration and removal, respectively. A variety of devices, e.g., Retisert^®^, Vitrasert^®^, Surodex™, and Ozurdex^®^ were designed and manufactured to be applied as ocular implants for therapeutic of illnesses with a chronic vitreoretinal nature [37]. A number of advanced delivery systems were designed to achieve higher ocular bioavailability, prolong drug release, and enhance patient compliance. In this context, drug releasing contact lenses can significantly extend the retention time on the ocular surface and could be loaded with nanoparticles that would improve the permeability of administered drugs. A major drawback for using this approach is frequent use of contact lenses which can be associated with corneal toxicity. Moreover, oxygen diffusion, microbial resistance, and effective and sustained drug release are yet to be addressed for successful clinical translation of this approach [12]. In the same context, cul-de-sac implants is a solid device placed in the conjunctival sac that could prolong drug release that would reflect on increasing patient compliance [38]. Punctum plugs are biocompatible devices inserted into tear ducts to block the drainage of tear fluid. They are non-invasive and can provide extended drug release to the anterior segment of the eye and could be formulated from non-biodegradable and biodegradable materials [12]. Ocular iontophoresis is a non-invasive method for active drug delivery using mild electric currents to enhance drug penetration through the ocular barriers. It can deliver drugs through *trans*-corneal route and transscleral route to the anterior and posterior segment of eyes, respectively [20]. Due to the novel developments of nanotechnology in recent years, ocular DDSs including nanoparticles [39], dendrimers [40], liposomes, nano-micelles, and other micro-/nanocarriers have also been widely used [41, 42]. Nanocarriers address many of the challenges to drug delivery to anterior and posterior segments through sustained and controlled release of the drug, protecting the drugs from ocular enzymes, and aiding in overcoming ocular barriers [39].

Commonly implemented delivery systems lack targeting to a specific ocular segment or tissue which may result in undesirable side effects to healthy tissues. Over the course of treatment, adjustment of drug dose may be required to be increased/decreased according to the progress of the disease/condition; however, most ocular DDSs release drugs at a predetermined rate cannot be adjusted according to the patient’s needs or changing physiological conditions [43, 44]. Consequently researchers have developed a variety of stimuli responsive DDSs (e.g., nanoparticles, hydrogels, and implantable materials) [45] that are stable when stored and transported and can effectively release drugs to the target sites after being stimulated [19]. Stimuli-responsive polymers are the focus of growing attention as they undergo physical or chemical changes in response to internal or external stimuli (examples of such stimuli include temperature, pH, ions, enzymes, and light [8, 19, 46]); and stimuli-responsive DDSs can control the release of drugs (e.g., increase the residence time of the drugs, promote drug targeting, and release the drugs on demand) [47–50]. DDSs responsive to exogenous triggers such as light are promising for clinical applications, as they are in part independent of the physiological conditions that may vary from one patient to another (e.g., enzymes and pH), and moreover, the release of drugs or therapeutic factors can be controlled by the intensity and duration of the external stimulation applied. The degree of adaptability and control makes external stimuli attractive candidates for on-demand DDS in personalized medicine applications [51, 52].

## Light-responsive drug delivery systems

Light is a promising method to remotely control drug release, especially in ocular DDSs. Irradiation of photoactive materials results in a variety of photophysical effects including photochemical (e.g., isomerization, cleavage, dimerization/polymerization, photosensitization [e.g., reactive oxygen species generation]) and photothermal effects; these are classified as reversible and irreversible changes. Such DDSs are particularly interestingly owing to the capability for precise spatiotemporal control, availability light sources of varying wavelengths, limited generation of side-products, convenience, and ease of use [8, 9]. Moreover, light can be applied with precision without the need for direct contact with the light-responsive DDS and does not need any additional reagent or solution components. Light can be easily switched on/off, and the rate of drug release can be optimized by tuning the wavelength, intensity, and duration of the irradiation, enabling precise control of drug delivery for specific applications [51, 53]. Light-responsive DDSs can reduce number of operations and can deliver therapeutic agents to the posterior eye segment in a minimally invasive manner. A triggerable drug delivery system would allow repeated on-demand dosing that would be adaptable to the patients’ regimen and allow multiple dosages from a single administration [43, 44]. On the other hand, ocular in situ gels are environmentally responsive polymers that are transformed from sol/gel with the small changes in specific conditions like pH, temperature, ionic strength, and light in the environment. Consequently, the residence time of the gel formed in situ will be prolonged, and the drug is released in an extended manner thus minimizing systemic absorption and reduced frequent dosing regimen [54]. Light could be used to trigger in situ gelation of drug-loaded polymers/monomers through crosslinking and polymerization sensitization [55]. In comparison with endogenous stimuli, e.g., pH, temperature, and ion, it enables precise structuring of the hydrogels (e.g., hardness and porosity) [19]. Similar to other exogenous stimuli, light offers more flexibility than endogenous stimuli, as it is less affected by different diseases or physiological variabilities which control the inter-/intra-cellular environment [56, 57].

Light-responsive DDSs have been developed utilizing UV, visible light, and NIR as sources of light, where the drug can be released using on/off sequences or completely released upon triggering, respectively [56, 58, 59], and are discussed in more detail hereafter.

### Photoisomerization

Photoisomerization is a reversible molecular conformational change around a restricted rotation site, usually a double bond, caused by irradiation with UV or visible light. In this context, compounds usually switch between the *trans* conformation and *cis* conformation, and azobenzenes and spiropyrans are the most investigated moieties that undergo photoisomerization reactions in DDSs to dynamically regulate the light-triggered drug release [9, 60]. A significant advantage of photo-responsive DDSs utilizing photoisomerization is that these moieties create a valve that can “turn on/turn off” drug release with good temporal control. These systems can also be used in single release systems [51].

#### Azobenzenes

**Fig. 4:**
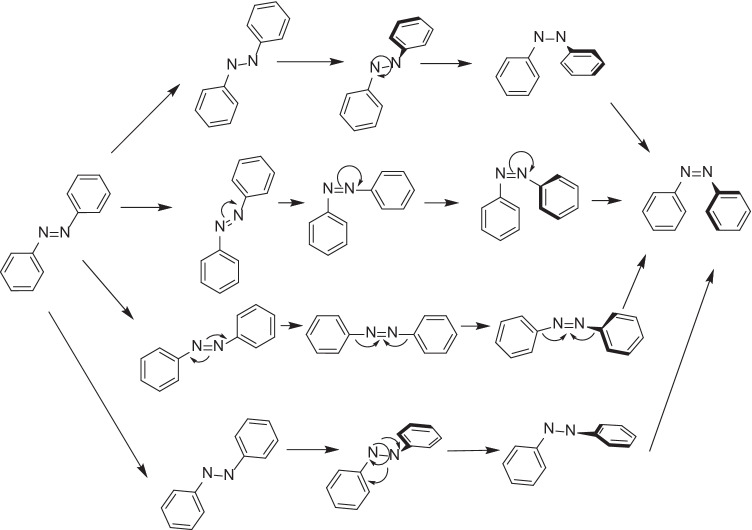
Mechanism of light-triggered photoisomerization of azobenzene

Polymers containing azobenzenes have a variety of interesting potential applications owing to their light-responsive nature [61]. Azobenzenes contain two phenyl rings that are interconnected through an azo group that responds to light and switches from the *trans* to *cis* conformation between wavelengths of 320 and 350 nm, with the reverse reaction occurring at 400–450 nm [62]. Photoisomerization of azobenzenes has been proposed via four potential mechanisms: rotation, inversion, concerted inversion, and inversion-assisted rotation (depicted in Fig. 4). In the rotational pathway, cleavage of the N = N π-bond allows free rotation around the N–N bond, which allows change of Ph–N–N–Ph dihedral angle, while the N–N–Ph angle remains fixed at ∼120°. Isomerization of azobenzenes by inversion involves one N = N–Ph angle increase to 180°, while the Ph–N = N–Ph dihedral angle remains fixed at 0°. Moreover, concerted inversion occurs when both N = N–Ph bond angles increase to 180° to produce a linear transition state. In inversion-assisted rotation, modifications in both Ph–N = N–Ph dihedral angle and N = N–Ph angles occur simultaneously [63–65].

Isomerization of azobenzenes is influenced by substituents through steric and electronic effects, resulting in significant changes to the absorption, emission, and photochemical properties of azobenzenes [61]. Substituents also have the ability to make the *cis* isomer more thermodynamically stable than the *trans* isomer [66–68]: for example, azobenzenes with amine derivatives at the 2 or 4 position, isomerization is achieved at a longer wavelength and occurs predominantly by inversion [69], whereas 2-hydroxy azobenzenes engage in tautomerization and H-bond formation which interferes with isomerization. The isomerization rates of different photoresponsive phosphate azobenzene derivatives, including varying lengths (mono-, di-, and tri-) and positions (ortho and para) of the phosphate groups, were investigated, observing that longer *o*-diphosphate and *o*-triphosphate azobenzene derivatives showed faster photoisomerization than those with shorter *o*-monophosphate groups; this observation could be ascribed to intramolecular interaction between the azo group and the phosphate groups which reduced the double bond characteristics of the N = N bond [70]. Thus, different AB derivatives may need validation in their release mechanisms and rates of photoisomerization. Inclusion of these moieties in a membrane-based system such as liposomes could disrupt the nanocarrier packing which releases the loaded drugs. This could be attributed to conversion of these chromophores to *cis* form upon photoexcitation, which is more polar/bulky and usually destabilizes these assemblies. Furthermore, the reversible nature of these reactions could modify drug release pattern to on/off and allow on-demand dose delivery. While these photoresponsive functional groups have been used in the development of DDSs, only a few studies have been performed in the eye [71]. A self-assembled cationic vesicle was formed from a cationic azobenzene derivative (4-cholesterocarbonyl-4′-(N,N,N-triethylamine butyloxyl bromide)-azobenzene) and the anionic surfactant sodium dodecyl sulfate in aqueous solution; rhodamine B (Model drug) was loaded into the vesicles. The system was administrated into rat retina by intravitreous injection to investigate the in vivo drug delivery behavior, and investigation of their release profile in vivo in rat retinas showed enhanced rhodamine release upon UV irradiation in comparison with control samples. Furthermore, fluorescence images of retinal sections showed the efficacy of the vesicles for drug delivery to rat retina and ability to preserve high drug concentrations for a longer duration [72]. The clinical usefulness of these systems is currently limited due to safety issues related to the use of UV-light and the potential toxicity of the chromophores [71].

Azobenzene photoswitches have been investigated for their potential to restore light sensitivity into retinal neurons after photoreceptor degeneration in disorders such as retinitis pigmentosa [73]. A potent photoswitch, BENAQ (an azobenzene bounded by a quaternary ammonium (QA) and a benzylethylamine), could pass through the plasma membrane of retinal ganglion cells (RGCs) and occlude the endogenous voltage-gated ion channels from the cytoplasmic side. Photoisomerization of BENAQ from *trans* to *cis* removes the blockade, and RGCs were able to depolarize and initiate action potentials. BENAQ isomerization from *trans* to *cis* could be accomplished by visible light irradiation and rapidly reversed in darkness [74]. The encapsulation of BENAQ into cyclodextrins [75] could alleviate the main challenge encountered with BENAQ delivery, as it precipitates near the injection site resulting in non-uniform photosensitization with a half-life of 7 days which is a transient period for therapeutic vision restoration. SBE-CD, a sulfobutylether β-cyclodextrin, forms a stable complex that increases solubility of BENAQ and improves photosensitization, extending light responses to a half-life of 31 days. This represents an exciting demonstration of the application of a supramolecular chemistry approach to drug delivery, in which the host–guest interaction between SBE-CD and BENAQ addresses a key challenge of intraocular drug delivery, guiding how photoswitches may be formulated as a possible approach for human blindness treatment in the future [75].

#### Spiropyrans

**Fig. 5:**
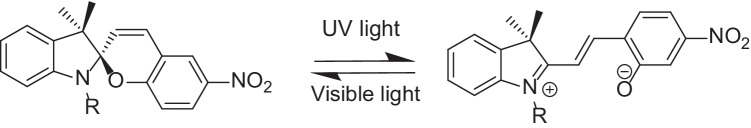
Mechanism of light-triggered photoisomerization of spiropyran

The reversible isomerization of spiropyrans (SP) is accompanied by a significant visible color change and is due to the different molecular properties of the two isomers of this molecule (spiropyran is the closed-ring isomer and is the more stable form (Fig. 5)). Spiropyrans consist of indoline and chromene moieties, which are conjugated together by a spiro junction and have a perpendicular spatial disposition in respect to each other. Light-triggering of this conformer results in cleavage of C–O bond and ring-opening followed by a *cis*–*trans* isomerization by rotation about the central C–C bond. Merocyanine has a planar structure and an extended π-conjugation between the chromene and indoline moieties; therefore, color changes may be observed upon transformation to this isomer (where SP usually absorbs light in the UV region); moreover, photoisomerization to merocyanine is accompanied by an increase in polarity that contributes to the disruption of the carrier’s stability [76, 77].

**Fig. 6:**
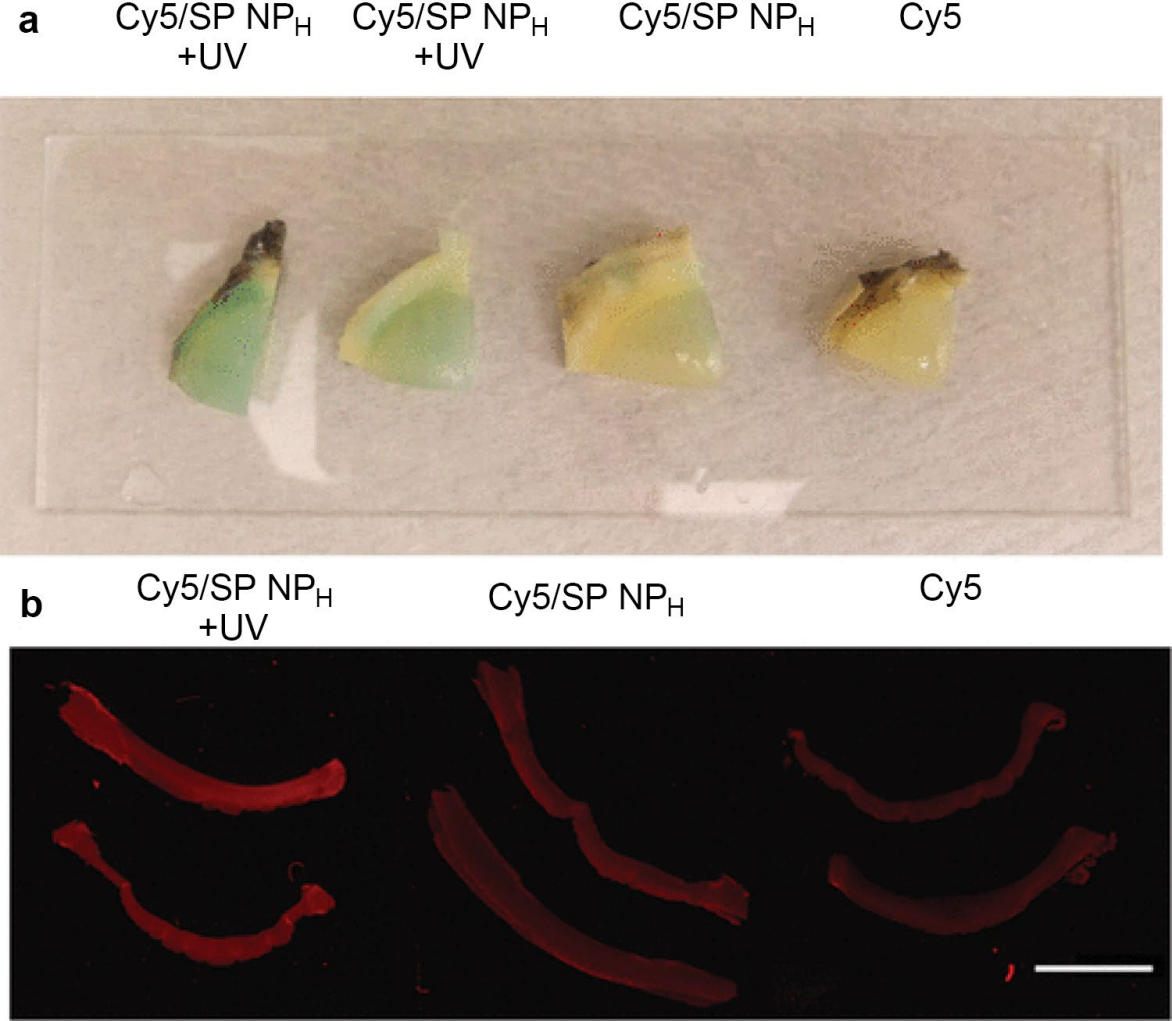
Ex vivo study of Cy5/SP NP_H_ penetration in porcine corneas. **a** Fresh corneas after an 8-h treatment with Cy5/SP NP_H_ (with or without UV irradiation for 1 min) or Cy5. The green color indicates the presence of Cy5 (a blue dye that becomes greenish in the slightly yellow tissue of the eye); **b** near-infrared images of cross sections of corneas tissues treated as in panel **a**. The scale bar = 1 cm. https://pubs.acs.org/doi/10.1021/ja211888a. Copyright 2012, American Chemical Society. Reproduced from [44] with permission. Further permissions related to the material excerpted should be directed to the ACS (support@services.acs.org)

UV light (365 nm) irradiation of self-assemblies of polymers incorporating spiropyrans with different carbon side chain lengths (C_7_, C_9_ and C_18_) led to isomerization of the spiropyrans, and for the polymers with the spiropyrans with C_9_ and C_18_ side chains a reduction in the size of the assemblies (not observed for the spiropyrans with C_7_ side chains) [44]. Size changes were explained by changes in polymer hydrophilicity after photoisomerization of SP to merocyanine, altering the microenvironment within the assemblies which was fully reversible for at least 4 continuous cycles (UV 365-nm irradiation for 30 s and visible light for 3 min). A hybrid SP/lipid-polyethylene glycol (PEG) NPs (termed NPH) were formulated to enhance the stability and loading efficiencies of NPs while maintaining the NPs’ photoswitching properties. The polymer self-assemblies were used to deliver Cyanine 5 (Cy5) to cadaveric porcine corneas with or without UV light irradiation, and visual inspection of the corneas and NIR scanning of corneal sections showed that irradiation with light enhanced the delivery of Cy5 represented by its green color distribution inside the tissues (Fig. 6). Histological examination of corneas for treated Cy5-loaded nanoparticles and UV light revealed no differences in comparison with untreated controls under light microscopy in terms of tissue injury, showing safety to corneal tissue. This is potentially advantageous for clinical applications, such as cancer treatment, where the tissue microenvironment can be leveraged to improve drug targeting and localize drug delivery to prevent the non-selective destruction of normal cells that cause the characteristic side effects of chemotherapy [44].

### Photocleavage

Light-induced cleavage of bonds can be applied to control the delivery of drugs, when, for example, a photolabile group (e.g., *o*-nitrobenzyl (ONB) and coumarin (CM) derivatives) is incorporated in a polymer backbone (and the drug is subsequently loaded into the matrix in a non-covalent fashion) or used as a photolabile covalent linker to conjugate drugs to the polymer, with subsequent irradiation of the resulting polymers with light-triggering drug release [57, 58].

#### o-nitrobenzyl derivatives (ONB)

**Fig. 7:**
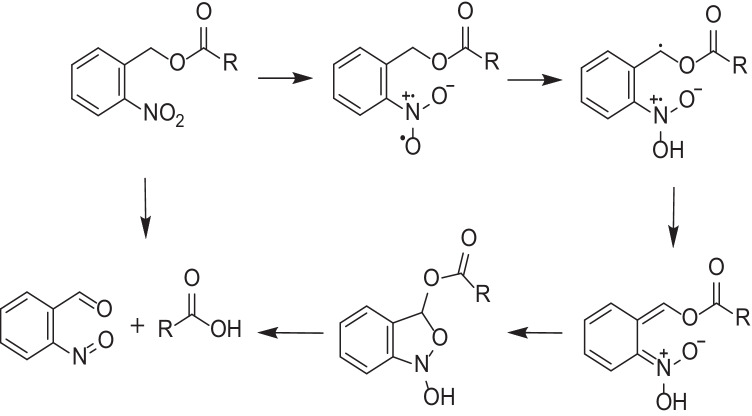
Mechanism of light-triggered photocleavage of ONB derivatives

Polymers containing light-responsive units are of interest for a variety of applications in materials science and engineering, with ONB derivatives being the most popular photocleavable groups [78]; ONB derivatives absorb a photon and form free radical intermediates, after which hydrogen abstraction occurs leading to a resonance-stabilized benzylic radical, and rearrangement of this species through a five-membered ring intermediate leads to the liberated carboxylic acid (drug) and a nitrosobenzaldehyde side product (Fig. 7) [79].

An interesting report of daylight-responsive drug-eluting contact lenses demonstrated the conjugation of timolol (a first-line drug for glaucoma treatment) to the contact lens matrix through a photolabile ONB derivative. When exposed to light of 400–430 nm, the timolol was released in vitro. Inhibition of intraocular pressure for up to a 10-h period was observed upon daylight irradiation of the lens in the treated mouse eyes. Results from an in vivo mouse model correlated with these findings and the contact lenses could effectively decrease the intraocular pressure of the mouse eyes; therefore, this approach could provide a promising platform for glaucoma treatment [80].

Intraocular lens implantation is a standard technique for cataract treatment, originally developed to reduce incidence of surgical complications. Light-responsive intraocular lenses have been developed to allow pulsatile drug release in a spatiotemporally controlled manner. Notably the repeated delivery of 5-fluorouracil in therapeutically relevant amounts of about 0.5–1.0 µg per dose was accomplished by the two photon absorption-triggered photocleavage of an ONB group in vitro [81].

**Fig. 8:**
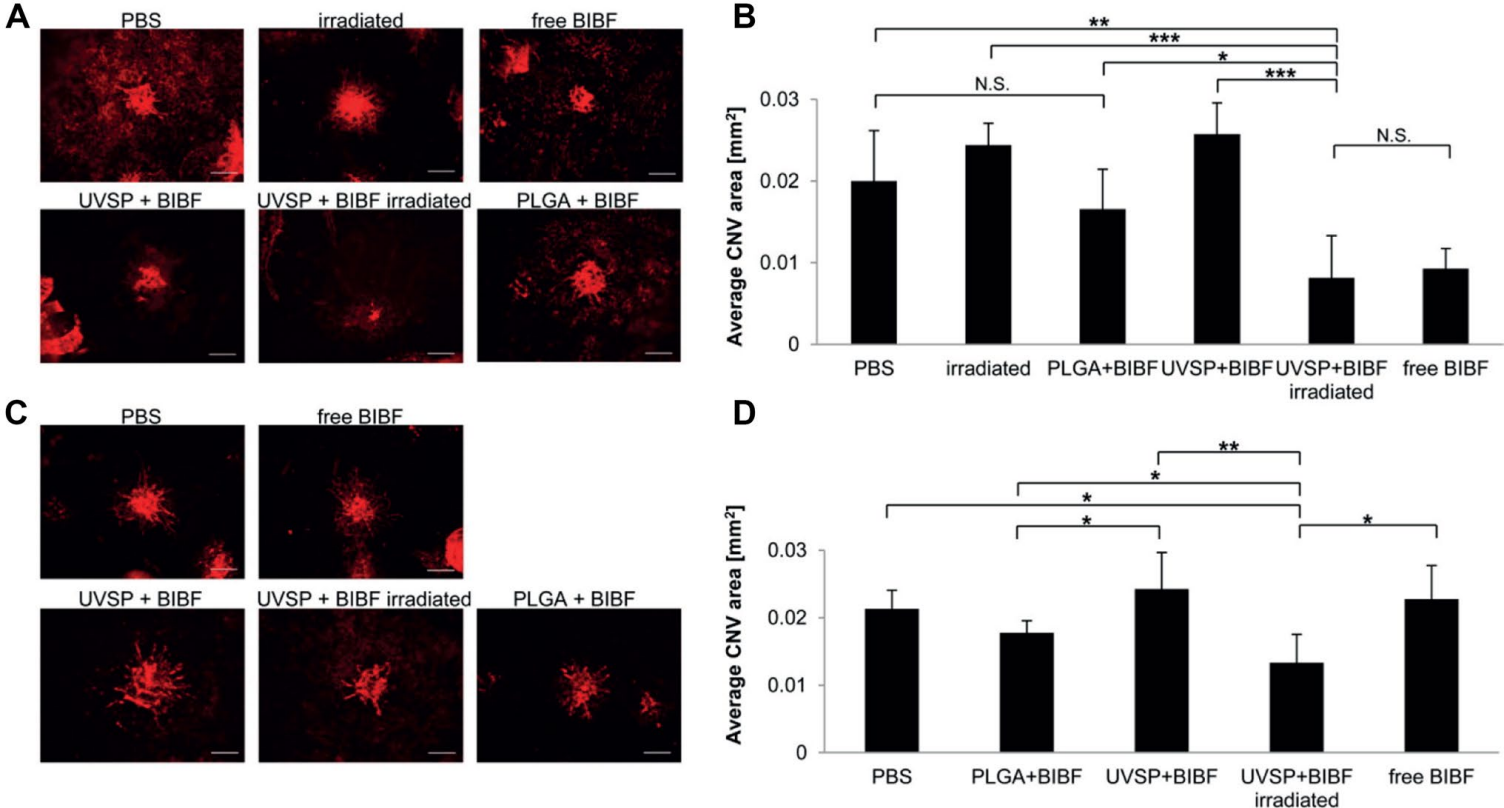
Light-triggered release of nintedanib (BIBF) post-injection inhibits CNV. **A** Fluorescent microscope images of isolectin B4-Alexa Fluor 594 stained choroidal flat-mounts 2 weeks after CNV induction. The eyes were irradiated immediately post-injection (scale bar = 100 µm). **B** Quantification of CNV spot size (*n* = 4–6). **C** Choroidal flat-mounts from the eyes irradiated 10 weeks post-injection, 2 weeks after CNV induction (scale bar = 100 µm). **D** Quantification of CNV spot size (*n* = 4–6). Copyright 2015, Elsevier. Reproduced from [82] with permission

Retinal diseases like age-related macular degeneration and diabetic retinopathy therapies may require repeated intravitreal injections, which limits patient compliance and increases the risk of infection and retinal detachment. Consequently, the need for non-invasive and controlled drug delivery to the posterior segment of the eye is significant [82, 83]. Nintedanib (a small molecule angiogenesis inhibitor that suppresses choroidal neovascularization) was loaded into light-responsive NPs based on copolymers employing an ONB moiety as a UV-labile photocage for a self-immolative polymer. Inhibition of vessel growth was compared to rats receiving PLGA particles containing nintedanib (BIBF 1120), free drug, or saline. Importantly, CNV areas in rats receiving UV light-released (365 nm, 5 min) nintedanib were significantly smaller than those in rats receiving PLGA-encapsulated nintedanib, suggesting that light-triggered release delivers drug more effectively than slowly hydrolyzing PLGA nanoparticles as depicted from Fig. 8. Injection of these DDSs into intravitreal fluid enabled on-demand delivery of nintedanib to mouse eyes in response to UV exposure up to 10 weeks post-injection [82, 83].

#### Coumarins (CMs)

**Fig. 9:**
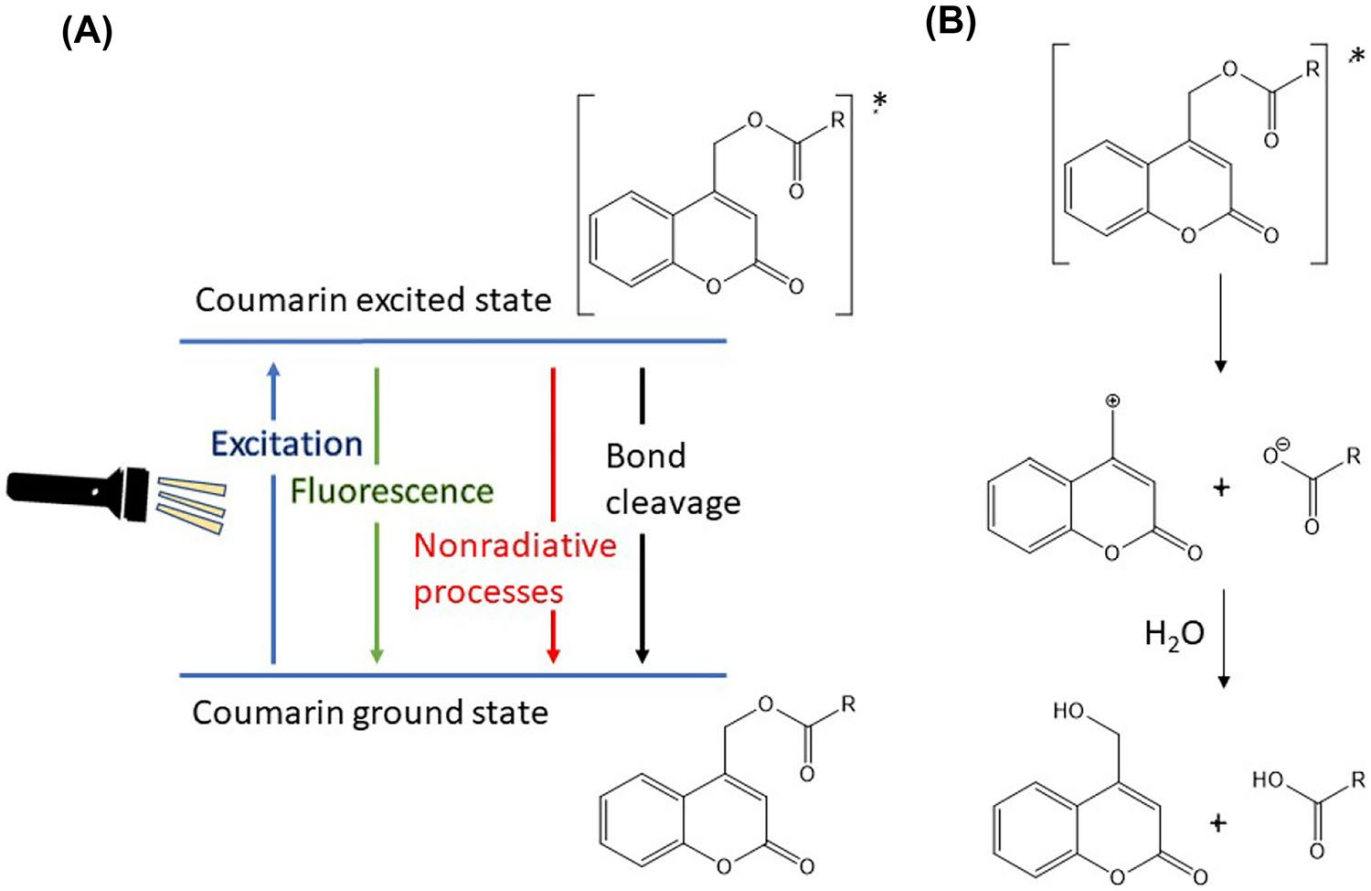
Photocleavage of CM/ligand conjugate. **A** Possible relaxation pathways for CM transition state. **B** Mechanism of CM/ligand bond cleavage

CMs are functional groups that can be incorporated into polymers designed to be light-responsive [84]. After absorption of a photon by CM-drug conjugates, relaxation to the lowest excited singlet state takes place, and deactivation of this transition state occurs by fluorescence and nonradiative processes and competes with heterolytic bond cleavage forming a singlet ion pair (CM^+^ and drug^−^). Product formation is proposed to happen in two steps: solvent separation of CM^+^ and drug^−^ ions, followed by the reaction of the CM^+^ cation with water, producing CM − OH and drug as depicted in Fig. 9 [85].

**Fig. 10:**
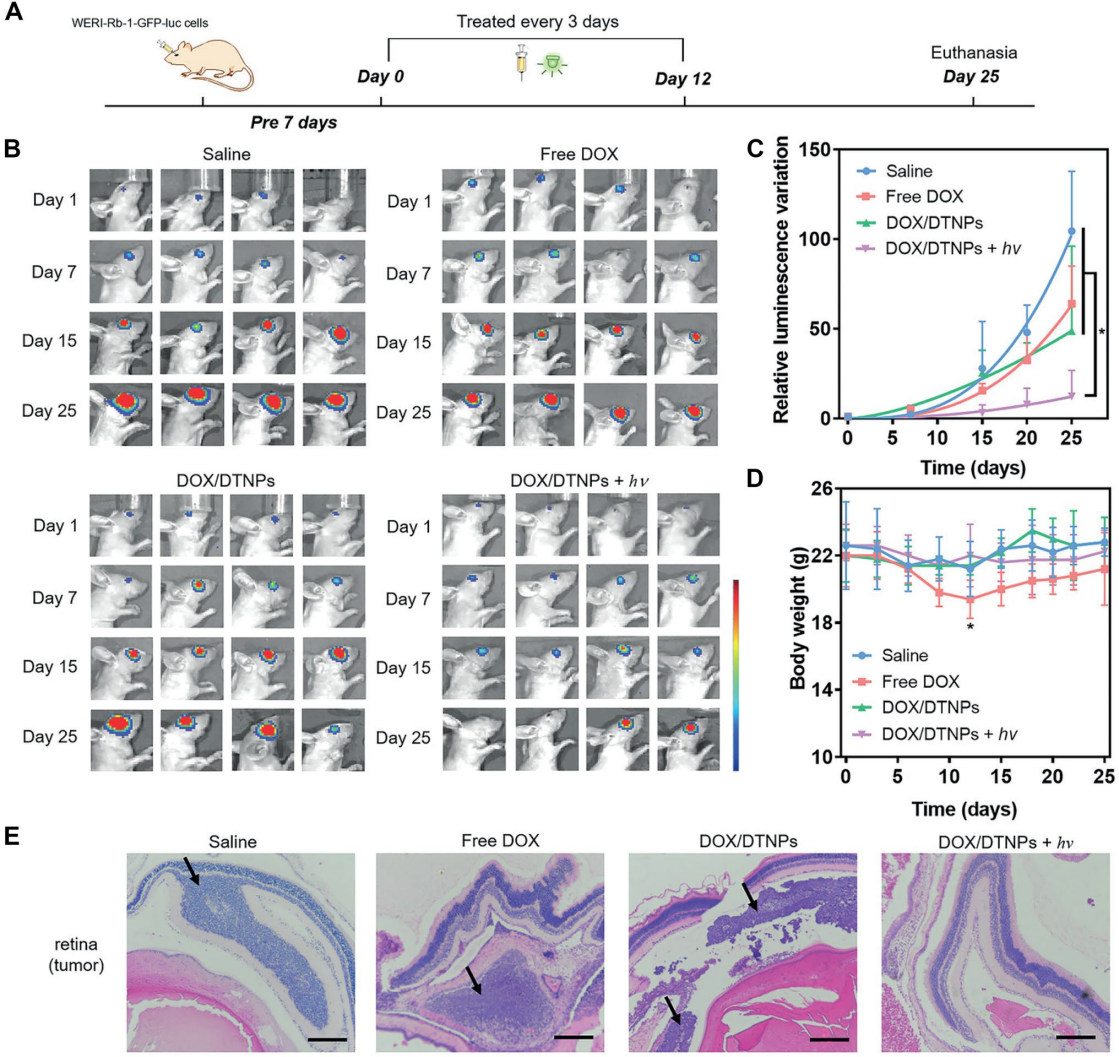
In vivo therapeutic effects of DOX/DTNPs. **A** Illustration of the procedures for the treatment of retinoblastoma. **B** In vivo bioluminescence images of the eyes in orthotopic WERI-Rb-1-GFP-luc tumor-bearing BALB/c nude mice on days 1, 7, 15, and 25. **C** Tumor growth curve presented by the intensity of bioluminescence of tumors after intravenous injection of various formulations. **D** Body weight changes of the mice in each group. Data were shown as means ± SD (*n* = 4). *p < 0.05. **E** H&E staining of the orthotopic retinoblastoma and retina tissue after different treatments. The scale bar is 200 µm. Attribution 4.0 International (CC BY 4.0). Copyright 2021, Wiley. Reproduced from [86] with permission

Light-responsive systems could enhance the efficiency of the systemic route in ocular drug targeting. Dicyanomethylene derivatives of CM which demonstrate green light responsiveness were conjugated to a trigonal core molecule (tris(2-aminoethyl) amine). This molecule can self-assemble into nanocarriers to achieve light-responsive drug accumulation in the eye for retinoblastoma treatment after systemic administration [86]. Meanwhile, surface PEGylation of the NPs was adopted to enhance their hydrophilicity, reduce immune clearance, and prolong the circulation time. DOX was loaded to the prepared NPs, and upon green light irradiation of the target eye, disruption of the NPs with subsequent drug release was demonstrated due to cleavage of the CM moieties. Retinoblastoma tumor model was established by injecting WERI-Rb-1 cells into the vitreous cavity of the right eyes of BALB/c nude mice for the tumor implantation. The tumor-bearing mice were intravenously injected with DOX/DTNPs and then treated with or without light irradiation. Tumor growth process was monitored by measuring the bioluminescence intensity on the day of the treatment (day 0) as the origin and determining the quantitative changes (compared to day 0) on the subsequent days (Fig. 10). On day 25, the increase of bioluminescence intensity in the eyes treated with DOX/DTNPs + UV (7.3-fold, compared to day 0) was significantly lower than those in the groups of saline (104.5-fold, compared to day 0), free DOX (64.0-fold, compared to day 0), and DOX/DTNPs (48.7-fold, compared to day 0). It should be noted that two of the mice treated with DOX/DTNPs + UV showed negligible bioluminescence signal on day 25, indicating that their tumors were almost eliminated. In addition, low systemic toxicity of the prepared system was demonstrated in vivo with reduced drug distribution to the heart, lung, liver, spleen, or kidney in the treated BALB/c nude mice [86].

A novel nanocarrier system consisting of light-activated cell penetrating peptides for choroidal neovascularization treatment was developed; the peptides were modified by 7-(diethylamino)coumarin-4-yl)methylcarboxyl groups which can inhibit cellular uptake in the blood after intravenous administration, which thereby facilitates the accumulation of nanoparticles composed of the peptide-CM derivative bioconjugate at the diseased choroid after being exposed to light (400 nm, 1 min). In a light-induced choroidal neovascularization mouse model, the neovascularization area in mice treated with light-activated doxorubicin-loaded nanoparticles was reduced by approximately 46%. Such light-responsive DDSs can be applied for the treatment of other conditions where light can reach the target sites, and effectively expanding the potential relies on the development of long wavelength light-triggered DDSs [87]. Moreover, a 5-fluorouracil prodrug was synthesized through its attachment to a CM by a [2 + 2]-cycloaddition reaction using UV irradiation typically above 300 nm in intraocular lens system for secondary cataract treatment. On the other hand, the cleavage of the cyclobutane linkage with subsequent drug release is triggered with higher-energy UV irradiation < 300 nm or using a multi-photon process. The controlled delivery of 5-fluorouracil was successfully showed in vitro; in addition, cell investigations confirmed the safety of the lens system [88–90]. The majority of research done on photocleavable triggered delivery systems has successfully fabricate delivery carriers that are biocompatible, able to efficiently encapsulate drugs, release them in response to light triggering, and achieve multiple release cycles with little-to-no leakage upon storage. However, light stimulation to photocleavable carriers usually creates irreversible changes; therefore, it fails to produce uniform release profiles from the carrier after each light exposure [91].

#### Other photocleavable moieties

**Fig. 11:**
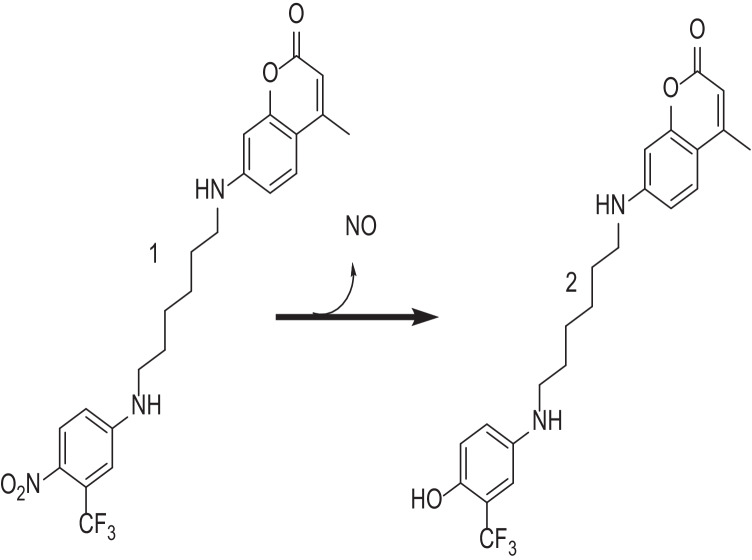
Schematic for the nitric oxide (NO) releasing chemical incorporated in contact lenses that release NO upon irradiation with visible/daylight

In another approach, visible/daylight assisted the activation of nitric oxide photodonors to reduce bacterial contamination of contact lenses [92]. Contact lenses were soaked in photodonor solution, and nitric oxide (NO) release was observed in response to visible/daylight irradiation. The photodonor is composed of a nitroaniline-based NO photoreleaser moiety linked, through an alkyl spacer, to CM, which facilitates the fluorescent detection of the NO release (Fig. 11). This could be explained by the blue fluorescence of the CM which is markedly quenched in the conjugate **1** by a Förster resonance energy transfer (FRET) mechanism. Excitation with visible/day light stimulates NO release from the nitroaniline moiety leading to the phenol derivative **2** as a stable byproduct. In comparison to conjugate **1**, FRET is not allowed in product **2**; as a consequence, the CM fluorescence emission is fully restored. This makes the NO release process easily monitored [93]. NO eluting contact lenses were well-tolerated by corneal epithelial cells with confirmed ability to induce growth inhibition of *Staphylococcus aureus* [92].

### Reversible crosslinking/de-crosslinking

**Fig. 12:**
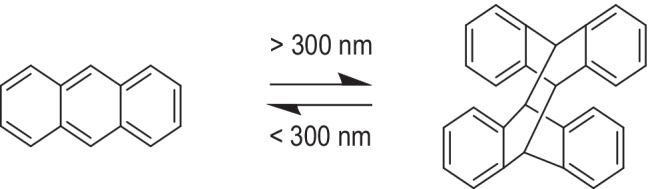
Photodimerization of anthracene

Photo-reversible dimerization reactions potentially enable crosslinking/de-crosslinking of polymers which has a variety of potential applications [94]. CM, stilbene, and cinnamic acid are commonly investigated moieties for [2 + 2] cycloadditions with one π-system being in an excited state, according to the Woodward–Hoffman rules to form cyclobutane rings [95]. The light-triggered dimerization of anthracene is accomplished through [4 + 4] photodimerization mechanism in which the photoexcited diene forms a short-life excimer that undergoes a transition into the cyclooctane structure [96]. Both additions occur upon irradiation with a light wavelength (> 350 nm) and were reversed upon irradiation with a UV light (< 260 nm). Indeed, employing photodimerization reactions facilitates the formulation of in situ gelling hydrogels with enhanced biocompatibility since they do not require photoinitiators and have fewer by-products [95]. For example, hydrogels were produced from polyethylene glycol-anthracene grafted hyaluronan for age-related macular degeneration treatment (Fig. 12). Anthracene grafted hyaluronan strands were crosslinked with UV (365 nm, 30 min) and were able to control the release of entrapped drugs according to UV exposure durations (e.g., Coomassie blue, fast green, and dextran; and lysozyme, bovine serum albumin, and myoglobin), demonstrating proof of concept that model drugs of various molecular weights could be delivered. The formulated hydrogels were compatible with human retinal cell lines and were shown to be enzymatically degradable (i.e., in the presence of hyaluronidase, an enzyme that is present in vitreous fluid) [97]. Implementation of photodimerization chromophores represent an alternative method of crosslinking the hydrogels and may extend drug release over prolonged periods depending on the degree of crosslinking; however, reversing the crosslinking reaction necessitates the use of light in the UV-B (280–315 nm) or UV-C (200–280 nm) regions which have poor ocular transmittance and potential biological complications owing to the wavelengths of light [98].

### Photopolymerization

**Fig. 13:**
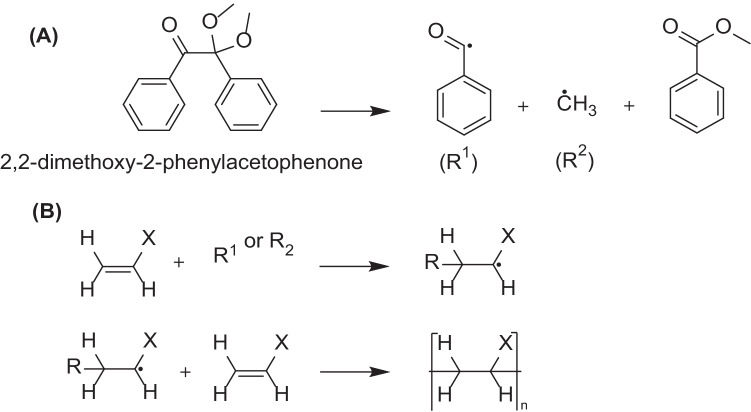
**A** UV-assisted 2,2-dimethoxy-2-phenylacetophenone cleavage into radicals. **B** General mechanism of photopolymerization reaction propagation

Photopolymerization is widely used in the development of polymer-based biomaterials [99]. Light-induced crosslinking is achieved by irradiating monomers in the presence of photoinitiator by a suitable light source. The light sources that have been evaluated in drug delivery, tissue engineering, and cell encapsulation were UV (330–450 nm) and halogen lamps (400–520 nm)[100]. Photoinitiators are moieties responsible for initiating polymerization reaction by producing reactive species (cationic, anionic, or free radicals) upon light irradiation e.g., eosin, 1-cyclohexyl phenyl ketone, and Irgacure 2959 [101]. Initiation of free radical production could be accomplished by heat or light irradiation. Propagation of the polymerization reaction is demonstrated by free radicals which attack on suitable monomers with subsequent generation of free radicals involved in further propagation and eventually termination (Fig. 13). Careful monomer selection enables control of properties like biodegradability, cell adhesiveness, and mechanical properties which would affect biomedical significance of the prepared polymers. Photopolymerization provides spatiotemporal control over the crosslinking process, fast curing rates at room or physiological temperature, and potentially being a non-/minimally invasive intervention [56, 102]. Light-activated crosslinking of polycaprolactone dimethacrylate and hydroxyethyl methacrylate for controlled delivery of bevacizumab was reported (employing the initiator 2,2-dimethoxy-2-phenylacetophenone and mixtures of polycaprolactone dimethacrylate and hydroxyethyl methacrylate). In vitro release results showed that the hydrogels were able to extend bevacizumab release for 4 months while maintaining its vascular endothelial growth factor-binding activity. After ocular injection of monomer mixture, photoinitiator, and light irradiation at 365 nm for 10 min, in vivo release of bevacizumab was detected in suprachoroidal space in rats for up to 2 months, and importantly, histological examination of ocular sections from treated animals revealed no morphological or structural changes which indicate the safety and nontoxic nature of the hydrogel [103]. The cytotoxicity of monomers and photoinitiators and the longer exposure to UV light in comparison to other photo-activated chromophores are the main limitations to fabricated hydrogels [23]. Therefore, the careful selection of the monomers, photoinitiator, and optimization of crosslinking intervals is mandatory to successful drug delivery using this approach. Implementation of light to trigger sol to gel change in ocular hydrogels provides many advantages over other stimuli-responsive systems receptive to a change in pH, temperature, and ions, e.g., their rapid sol–gel transformation resulting in better mechanical properties [2]. Moreover, the transparent nature of cornea and lens facilitates light penetration to the posterior segment in a non-invasive manner. Therefore in [104], peptide (connexin43 mimetic peptide as a model drug)-loaded PLGA NPs were incorporated to methacrylated alginate, and light was used to trigger its gelation for implantation in the vitreous body. Photo-crosslinking demonstrated a reduction in the hydrogel porosity and would prevent the NPs free movement and thus rapid elimination from the vitreous fluid. The sulforhodamine B assay and zebrafish embryo toxicity model indicated the biocompatible nature of the loaded NPs and crosslinked hydrogel which is a prerequisite for drug delivery to a sensitive organ such as the eye.

In the case of liposomes, engineering their surfaces and bilayers is mainly aimed at enhancing the delivery of transported cargos and their controlled release onto targeted sites (in vitro or in vivo), for therapeutic or other purposes. Light-controlled drug delivery from liposomes was accomplished by number of approaches; photo-crosslinking within the hydrophobic domain of the lipid bilayer causes regions within the bilayer to act as pores for enhanced drug release. Light responsiveness could be conferred by including moieties such as dienoyl, sorbyl, and styryl, which are responsive to short wavelength UV light. Light-responsive lipids such as bis-dienoyl-phosphatidylcholine or 1,2-bis[10-(2′,4′-hexadienoyloxy)-decanoyl]-*sn*-glycero-3-phosphocholine, each has an activated diene group incorporated in the lipid tail near the glycerol backbone or at the end of the hydrocarbon tail, allow the light-induced formation of crosslinked polymer networks [105, 106]. Moreover, incorporation of an effective sensitizer dye 1,10-dioctadecyl-3,3,30,30-tetramethylindocarbocyanine to the liposomes could further render them responsive to higher wavelengths of light (e.g., green light) [107]. In the same context, photopolymerizing polymers (e.g., methacrylated polyethylene glycol derivatives) could be incorporated into the liposomal bilayer to enhance its permeability [108].

### Photosensitization

**Fig. 14:**
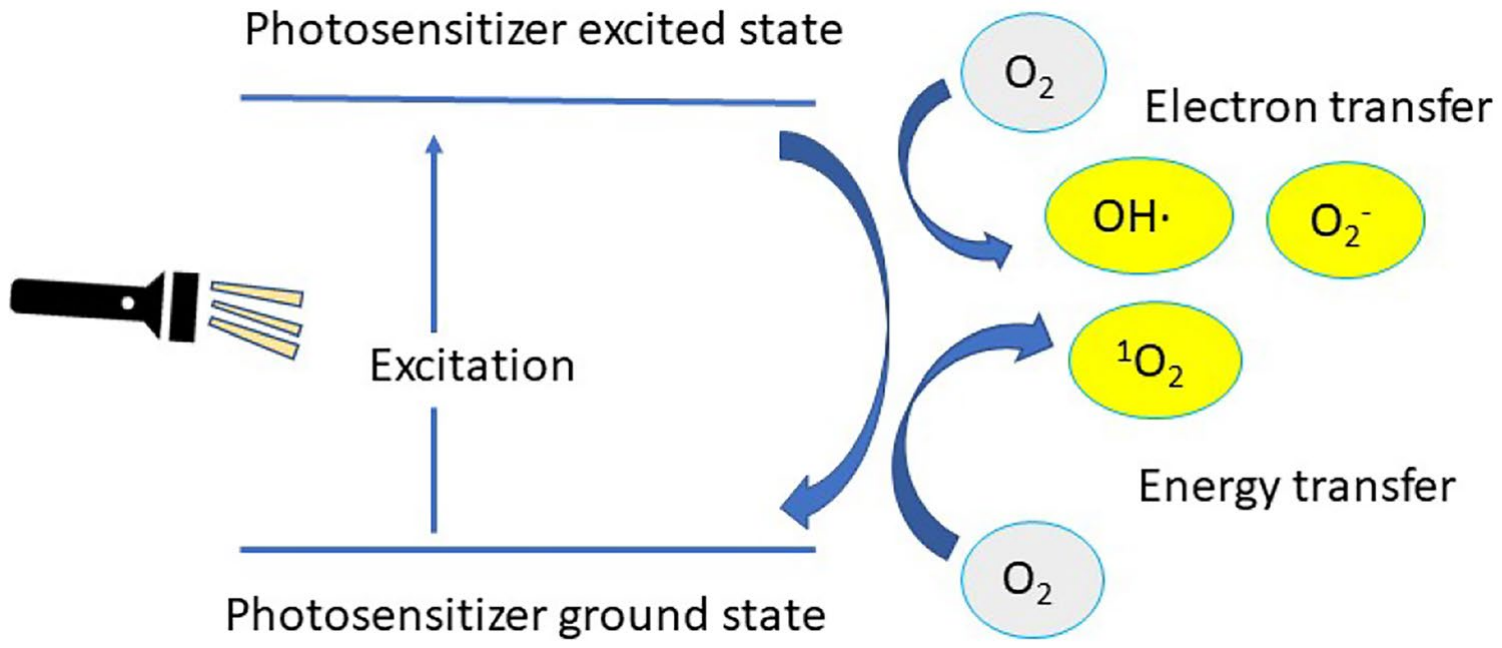
General mechanism of action for photosensitizers

Photosensitizers are molecules which absorb light and transfer the energy from the incident light into another nearby molecule by energy or electron transfer [109]. It induces the production of reactive oxygen species such as singlet oxygen (^1^O_2_), hydrogen peroxide (H_2_O_2_), superoxide anion (O_2_^·^-) and hydroxyl radical (OH^.^) upon illumination of a sensitizer moiety (Fig. 14). Photosensitizers could be classified according to their chemical nature into organic photosensitizers (which are sub-classified to synthetic dyes (e.g., phenothiazinium, indocyanine green, hematoporphyrin, and boron-dipyrromethene), natural compounds (hypericin and riboflavin), and tetrapyrrole derivatives (e.g., porphyrins and phthalocyanines)) and inorganic photosensitizers (e.g., TiO_2_ and ZnO) [109–112]. This technique is currently used in photodynamic therapy that involves using a photosensitizer coupled with an appropriate light source in the presence of molecular oxygen. The photo-mediated activation of the photosensitizer results in reactive oxygen species generation, leading to the destruction of the target tissue or cells by apoptosis to disrupt the membrane of cancer cells and induce cell death [113–115]. Furthermore, photodynamic therapy has been evaluated in treatment of ophthalmic disorders like age-related macular degeneration and transplant rejection [116, 117]. In this context, Visudyne is a US Food and Drug Administration-approved verteporfin (a benzoporphyrin-derivative photosensitizer)-loaded liposomal formulation that reduces the risk of vision loss addressed by age-related macular degeneration. Clinical use of this drug product is facilitated by its intravenous infusion using sterile water for injection followed by the application of non-thermal red light. In addition, verteporfin combination with anti-vascular endothelial growth factor therapeutics and steroid drugs like triamcinolone acetonide demonstrated superior efficacy in decreasing the frequency and number of treatment sessions and improved visual prognosis [118, 119]. In the same context, loading of a dendritic porphyrin into polyethylene glycol-block-poly(L-lysine) micelles was accomplished in an attempt for age-related macular degeneration treatment. The characteristic dendritic structure prevents aggregation of its core sensitizer, thereby inducing a highly effective photochemical reaction. It showed superior accumulation into diseased lesions in comparison with free dendritic porphyrin. This resulted in a superior efficacious CNV occlusion with minimal phototoxicity [120]. Light-induced oxidation was implemented in developing light-controlled release of therapeutic agents from oxidizable nanocarriers/endosomes. This was accomplished by irradiation of photosensitizer which will promote leakage of encapsulated cargos by disrupting the carrier/endosome membranes. Plasmid DNA polyplexes (composed of plasmid DNA in conjunction with cationic peptide which contain a nuclear localization sequence that would promote gene transfection to the cells) were loaded into phthalocyanine dendritic photosensitizer carriers for ocular gene delivery, and upon light irradiation, the engulfed phthalocyanine nanocarriers will damage the endosomal membrane, thereby, promoting endosomal escape of the plasmid DNA polyplexes and allowing gene nuclear delivery. Wistar rats were given a subconjunctival injection of dendritic photosensitizer/polyplex and irradiated using a 689-nm laser; the results showed that the formulated system was able to enhance gene expression in vitro and in vivo with reduced phototoxicity. The DDS could be applied to a targeted gene carrier for the treatment of various diseases, including solid tumors. Spatial control of gene expression in the body will ensure the effectiveness and safety of in vivo gene therapy [121]. Photochemical triggering usually induces an irreversible change in the delivery system, which means that this approach is useful for single burst drug release applications not for gradual and repeated pulses of drug release. It is desirable to use this strategy when destruction of the target tissue is desired, e.g., cancer cells and age-related macular degeneration treatment. However, it would not provide a satisfactory approach when only drug delivery and targeting is desired.

### Photothermal reactions

**Fig. 15:**
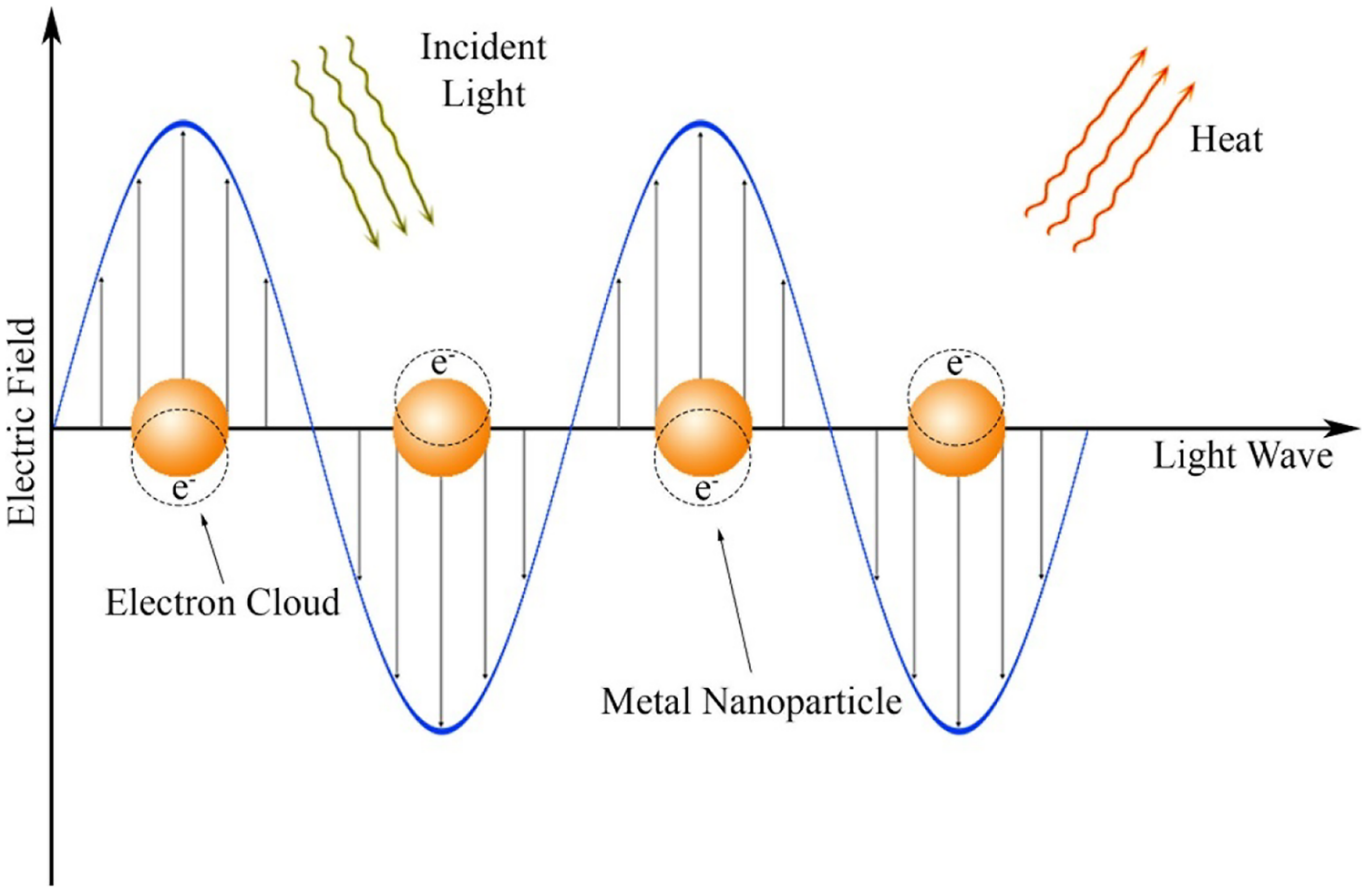
Visualization of the localized surface plasmon resonance of metal nanoparticles–a collective oscillation of valence electrons, in response to an oscillating electric field of light at the resonance frequency. Stimulation of the plasmon by light causes localized heating in the vicinity of the metal nanoparticle. Copyright 2021, Elsevier. Reproduced from [60] with permission

Photothermal reactions have been explored for ocular drug delivery through the interaction between a plasmonic material, light activation, and thermally sensitive drug carrier. Gold (Au) nanoparticles are able to rapidly absorb energy from UV, visible, and NIR sources and release energy as heat within picoseconds. This could be achieved if the incoming light wavelengths match the Au nanoparticles localized surface plasmon resonance absorption bands; this would result in the generation of a hot electron gas (i.e., a collective oscillation of conduction band electrons produces energetic plasmonic electrons). This rapidly loses energy by heat exchange within the particle’s surroundings (Fig. 15). Accordingly, light-absorbing materials such as Au nanoparticles embedded in DDSs can absorb incident photons and transform it to thermal energy which may rupture the carrier and subsequent payload release, and it is noteworthy that Au nanoparticles are widely used due to their bioinert/nontoxic nature in addition to tailorable optical and photothermal properties [122–125].

**Fig. 16:**
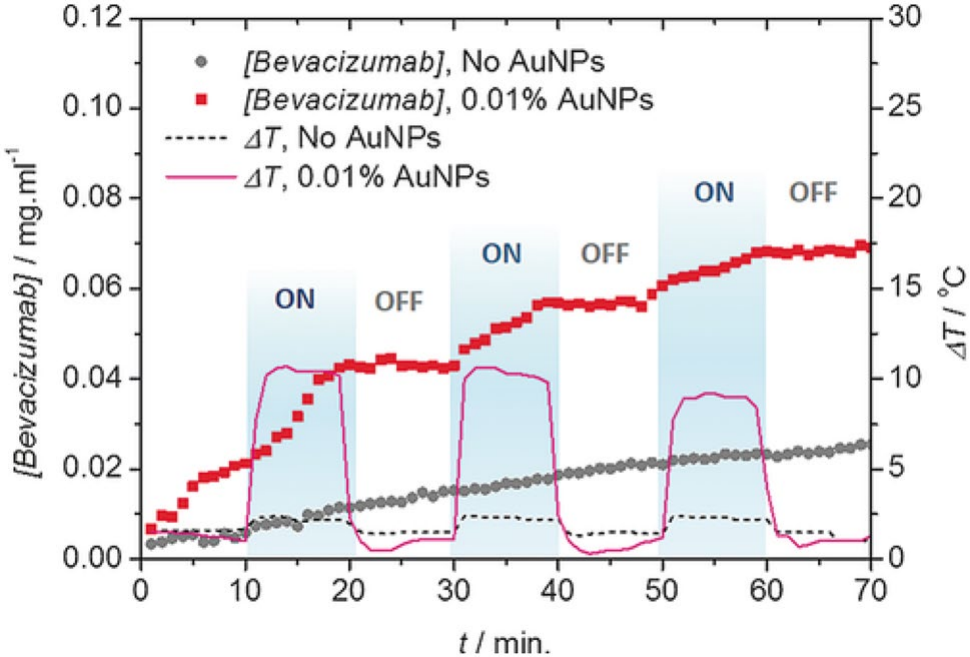
Photo-modulated temperature increase and release of bevacizumab or Avastin^®^ from a hydrogel depot (1.25 mg mL^−1^ [bevacizumab] + 2% w/w agarose) loaded with 0.01% w/w AuNPs (0.1 mg mL^−1^ AuNPs) and a control depot without AuNPs. The hydrogel depots were exposed to blue light (508 mW cm^−2^, 400–500 nm) for 10 min three times. Copyright 2016 Wiley. Reproduced from [126] with permission

A light-triggered hydrogel for the controlled delivery of bevacizumab was composed of AuNPs embedded in agarose gels that was designed for treatment of age-related macular degeneration [126]. This was achieved by irradiating bevacizumab-loaded hydrogels with visible light (400–500 nm, 10 min) for successive cycles. The AuNPs used in this system perform photo-thermal conversion to increase local temperature of the hydrogel. The temperature change triggers a sol–gel transition and drug diffusion to the surrounding environment. The release of drug follows an on/off cycle with light irradiation (Fig. 16), wherein the AuNPs/hydrogel system was able to enhance drug release by three-fold in comparison with hydrogels without Au nanoparticle. The released levels of bevacizumab were implemented to demonstrate the efficiency of the photo-triggering. However, investigation of bevacizumab at lower concentrations demonstrated efficiency in blocking the activity of human vascular endothelial growth factor [126]. In a similar approach, Au nanorods (AuNRs) were loaded into chitosan/puerarin hydrogels as a treatment modality for uveal melanoma (UM) [127]. The incorporation of AuNRs could regulate the mechanical strength of the hydrogel, and their photothermal properties could facilitate sol–gel transformation to release the loaded drugs (e.g., gene-targeted anticancer drug DC_AC50) on demand (on/off cycles) in response to NIR irradiation. Drug-loaded hydrogel was injected into the eyeballs of mice (orthotopic model of UM), and results demonstrated the superior efficacy of the hydrogel in suppressing tumor growth without causing damage to normal tissue, which could be attributed to synergistic effect of photothermal therapy of AuNRs and gene-targeted therapy. The antibacterial properties of hydrogel (chitosan/puerarin) in addition to gene-targeted therapy/photothermal treatment provide a promising strategy for building multifunctional therapeutic platform against intraocular tumors and exhibit great potential for the clinical translation for UM treatment. In the same context, AuNPs were loaded to a dual-responsive liposomes (heat-/pH-responsive), which can achieve selectivity in the acidic compartment (endosome, lysosome) in cells when triggered by visible and NIR light [128]. Similarly, AuNRs were able to trigger phase transition in phospholipid-based nanodroplets with on-demand delivery of DOX [129]. In another study, a NIR-responsive liposomal platform for on-demand delivery of urokinase-plasminogen activator (uPA) using a hybrid formulation of ultrasmall AuNRs, thermosensitive phospholipid (DPPC) and non-ionic surfactant (Brij58) was reported. This DDS was investigated for bleeding-free photothermally assisted thrombolysis, where the synergistic photothermal effect of AuNRs and the released uPA would facilitate clot lysis. uPA-loaded liposomes showed 80.7% (± 4.5) lysis of an in vitro halo-clot model in 30 min following NIR irradiation (785 nm, 1.35 W/cm^2^ for 5 min) compared to 36.3% (± 4.4) and 15.5% (± 5.5) clot lysis from equivalent free uPA and non-irradiated liposomes, respectively. These results show the potential of low-dose, targeted thrombolysis via the combination of light-triggered delivery/release of uPA from liposomes combined with photothermal thrombolytic effects from AuNRs [130]. Due to their photothermal properties, AuNPs were investigated in ocular cancers treatment alone [131] or in combination with anticancer agents [132]. In the same context, near-infrared (NIR) fluorescent dyes have been investigated in ocular drug delivery, whose emission wavelengths are between 740 and 1700 nm, due to their minimal side effects, high photothermal conversion, and long absorption wavelength. The structures of fluorescent dyes mainly consist of cyanines, phthalocyanines, BODIPYs, and rhodamine analogues, of which indocyanine green (ICG) has been approved by the FDA [133]. Silicon 2,3-naphthalocyanine bis(trihexylsilyloxide) was used as a photothermal moiety for triggering light-responsive lipid-based carriers as a potential DDS for age-related macular degeneration treatment [134]. Indocyanine green was loaded into hyaluronic acid-coated liposomes as a light activating moiety for the controlled intravitreal delivery of calcein; the liposomes prepared showed adequate stability and mobility in vitreous fluid, and longer irradiation periods demonstrated enhancement of calcein release [135]. In another approach, ICG was assembled in liposomes for photothermal therapy of retinoblastoma, and the liposomes were able to overcome limitations usually encountered with ICG delivery, e.g., quenching, aggregation, and instability. Moreover, the liposomes could achieve specific tumor tissue targeting and penetration in vitro and in vivo and efficient accumulation in solid tumors which enhanced the efficacy of tumor photothermal therapy [136].

NIR responsive drug delivery systems were fabricated for chemo-photothermal therapy of posterior capsule opacification [137]. The main modality for cataract treatment is by replacing the diseased lens with an intraocular lens, which initially restores high visual quality. Unfortunately, posterior capsule opacification, which can often occur after contemporary cataract surgery, is likely to cause the second loss of vision. In this approach, doxorubicin was integrated into black phosphorus nanosheets onto the non-optical part of commercial intraocular lens, for chemo-photothermal combination therapy of posterior capsule opacification. Taking advantages of the large surface area and negative charge of black phosphorus nanosheets, black phosphorus nanosheet dispersions were used as nanocarrier biomaterials for photothermal therapy and loading efficiency 847% w/w for positively charged doxorubicin. This system was characterized by good transmittance, robust mechanical properties, NIR dual-triggered drug release behaviors, and excellent photothermal efficacy. Finally, chemo-photothermal combination therapy studies of intraocular lens system in vivo were also investigated using rabbit models. The results showed that NIR irradiation group exhibited distinct superiority on the aspect of inhibiting residual lens epithelial cells growth, and this system could be applied as a promising strategy for chemo-photothermal synergistic treatment for posterior capsule opacification [137].

Au nanoparticles are generally well tolerated in short-term administration, but their long-term effects and ocular safety have not been proven. Their biocompatibility or toxicity was observed according to the AuNPs shapes and sizes, their ligands, the species on which the studies were performed, and the doses used [138], where metal nanoparticles 20 and 80 nm in diameter were toxic to photoreceptor cells in vitro [139]. Moreover, AuNPs (with a diameter between 5 and 30 nm) and AuNRs (with 90 nm length and 10 nm diameter) lead to more cytotoxicity in comparison with larger NPs on adult retinal pigment epithelial cell line 19. The internalization efficiency was also influenced by the diameter of AuNPs, and only the ones with a diameter smaller than 30 nm were highly internalized (more than 86%) [138]. On the contrary, indocyanine green proved its safety over the last decades for its clinical use as imaging agent [133]. The considerable amount of heat generated by this method could be detrimental to the surrounding tissues. Additionally, this strategy is perhaps most limited by the availability of thermally responsive materials that are both biocompatible and demonstrate a robust thermal response at physiologically relevant temperatures [140].

## Challenges

**Fig. 17:**
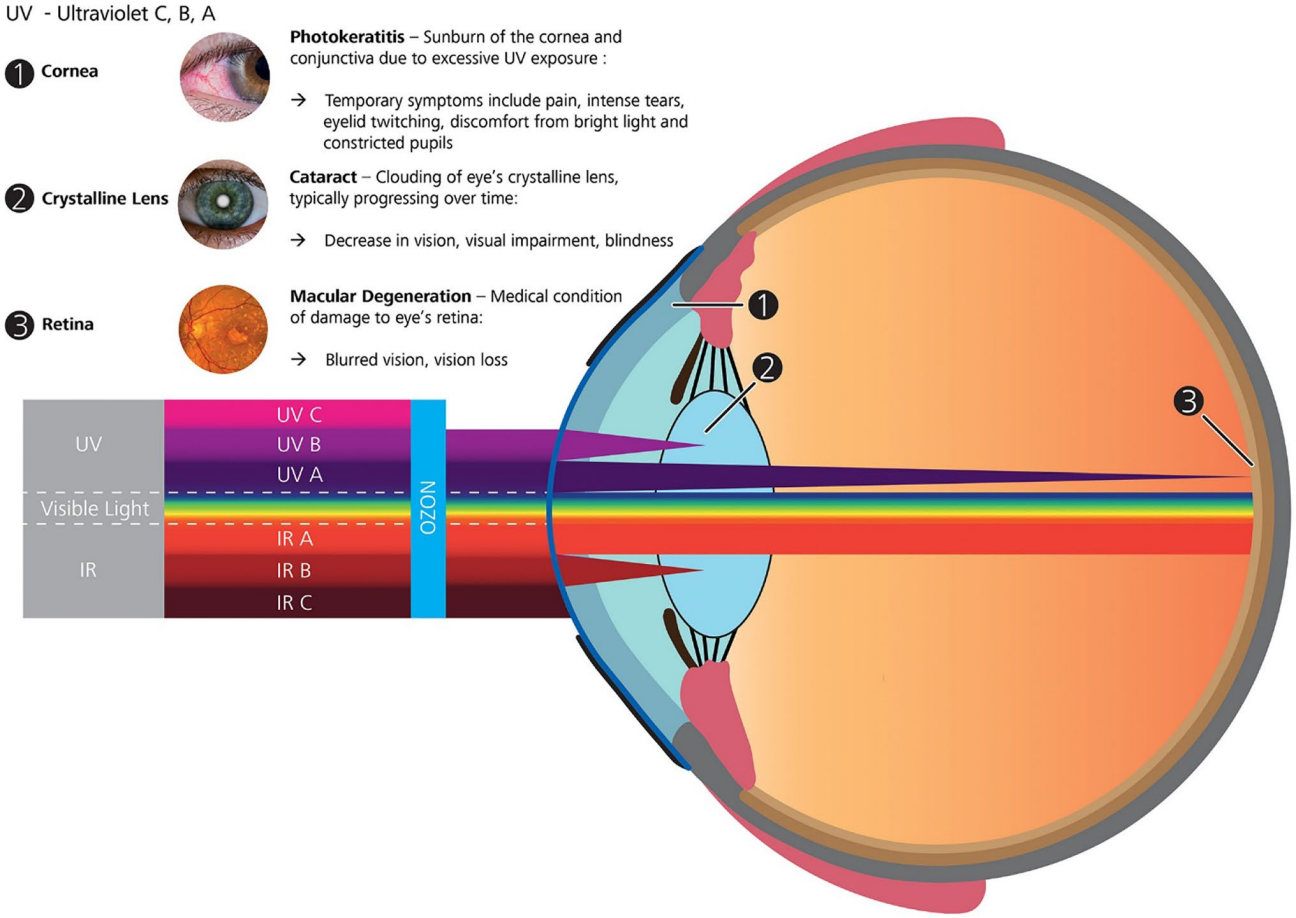
A schematic diagram of the eye showing the relative propagation of the different optical radiation bands through the ocular tissue. The optical media (cornea lens, aqueous humor, and vitreous humor) is generally transparent only to wavelengths in the visible and IRA bands. UVC and UVB are mostly absorbed by the nucleotide bases and aromatic amino acids and therefore do not propagate past the cornea and the lens, respectively. The IR bands beyond 1400 nm (IRB and IRC) are increasingly absorbed by water molecules and do not penetrate past the superficial cornea. UVA and UVB radiation reaching the retina varies with age, but it is estimated that in adulthood less than 2% UVA and 1% UVB radiation (not illustrated) reaches the retina. Under certain circumstances, the different structures of the human eye and the retina may be damaged by solar or coherent laser radiation (reprint). Attribution 4.0 International (CC BY 4.0). Copyright 2018, Wiley. Reproduced from [141] with permission

Light-responsive biomaterials present a variety of potential advantages for DDSs targeting the anterior and posterior eye compartments; however, ocular light transmittance and the safety of the light source are the main challenges to their successful development. Light transmittance in the eye is wavelength dependent as shown in (Fig. 17) [141]. Limited ocular transmittance and cytotoxicity are the major disadvantages of UV radiation (100–400 nm) [142]; nevertheless, UV-A (315–400 nm) is able to penetrate the entire lens and reach the retina and is therefore suitable for the development of light-responsive ocular DDSs [71]; NIR (800–1100 nm) has superior tissue penetration up to several centimeters and lower absorption by water and lipids. The damage mechanism of light depends on its wavelength, intensity, and the irradiation time. Three main types of tissue light damage are distinguished: thermal (inflammatory response), photomechanical (stress confinement), and photochemical (photo-oxidation). Photothermal and photomechanical damage are associated with NIR exposure, while photochemical effects are mainly associated with exposure to UV light [141]. Light with a wavelength of 365 nm and intensity of 5.4 mJ/cm^2^ showed no cytotoxicity to corneal cells in vitro and in vivo [143]. Generally longer wavelengths have higher safety profiles to cells and tissues due to their lower energy [144–146]; consequently, NIR irradiation is preferred to UV light for a variety of in vivo applications and could be implemented in inducing DDSs implanted in the vitreous body [147], however, most NIR-responsive chromophores require longer irradiation times that may result in burst release. Significant effort has been directed to develop NIR-responsive chromophores that respond rapidly by utilization of non-linear photon excitation, such as two-photon absorption and upconversion. In this context, NIR irradiation could be converted to higher energy photons needed for activation of light-induced reactions while preserving deep tissue penetration and minimal phototoxicity to the tissues [58, 59]. Upconversion nanoparticles are NIR-absorbing materials (commonly rare earth inorganic nanomaterials doped with lanthanide ions) that can generate higher energy photons such as UV or visible light using low energy photons from the NIR spectral region functioning via excited state absorption and energy transfer upconversion. The upconversion mechanisms and their applications in drug delivery and biomedical applications have been summarized in excellent reviews [59, 148–151]. However, there is a lack of standardized protocols for the toxicology assessment of UCNPs, and it is difficult to critically evaluate the results from various investigations because the cytotoxicity studies utilized different conditions (time of exposure and dose) using NPs with varying morphology, chemical composition, size, surface charge, or functional groups, and on various cell lines [152]. In two photon absorption, a light-responsive molecule is excited to a higher energy state by simultaneous absorption of two photons from the incident light to produce a higher energy photon equal to the sum of the energies of the two photons. However, triggering a photoresponse by two photon absorption requires irradiating DDSs with high intensity lasers for long intervals which limits the safety and applicability of this approach [59]. Most of the light-responsive systems have been used for the delivery of small molecule drugs; however, exploration of the efficacy of different systems in delivery of peptides, proteins, and DNA and RNA for gene therapy need to be adopted [51]. Despite these challenges, light-responsive DDSs have significant prospects in treating ocular disorders and require further investigation to generate DDSs capable of exploitation to release drugs with spatiotemporal control yielding the smart ophthalmic DDSs of the future.

## Future perspectives: towards clinical applications

**Fig. 18:**
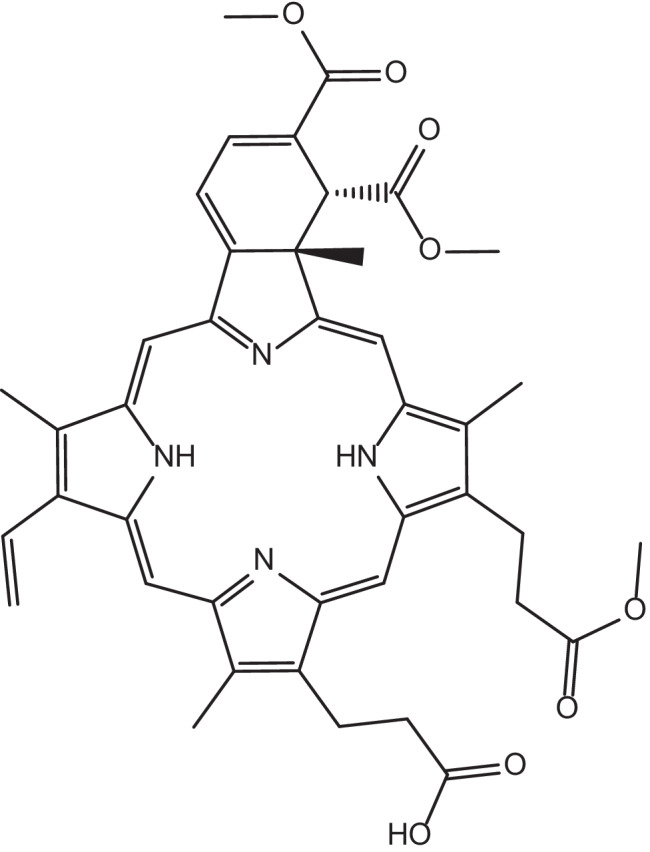
Chemical structure of verteporfin

The focus of the preceding sections was preclinical studies involving light-responsive DDSs for ocular applications, and this section briefly summarizes pertinent clinical trials supporting progress towards clinical applications of such DDSs. The clinical use of photodynamic therapy is facilitated through Visudyne^®^, a US Food and Drug Administration-approved light-responsive delivery system. Visudyne is a liposomal formulation that reduces the risk of vision loss in cases of subfoveal choroidal neovascularization due to age-related macular degeneration or subfoveal choroidal neovascularization secondary to pathological myopia, particularly in the absence of occult choroidal neovascularization [153]. The photosensitizer verteporfin (Fig. 18), a benzoporphyrin-derivative monoacid ring A, is the active ingredient of Visudyne^®^. The finished drug product is a green, lyophilized liposome powder that is reconstituted before intravenous infusion using sterile water for injection. Visudyne^®^ is administered in a two-stage process requiring both the injection of verteporfin, followed by the application of non-thermal red light after a 15-min interval (exposure time of 83 s at a dose of 50 J/cm^2^ of neovascular lesion, at a light intensity of 600 mW/cm^2^ at 689 ± 3 nm wavelength (non-thermal red light) using a diode laser) [92].

A series of clinical trials have implemented photodynamic therapy for the treatment of ocular conditions with proven efficiency. Periocular basal cell carcinoma was treated with topical aminolevulinic acid photodynamic therapy combined with surgery (after removal of the tumor, each tumor region was irradiated with 177 J/cm^2^ using a 635-nm laser for 15 min; a total of 3 times of assisted aminolevulinic acid photodynamic therapy was applied during and after operation). Aminolevulinic acid photodynamic combined therapy reduced the need for delicate surgery for tumor removal, enhanced rapid rehabilitation, preserved the function of eyelid, and decreased the probability for disfigurement and scar tissue formation [154]. Furthermore, the effect of an indocyanine green-enhanced laser (810 nm) thermocoagulation method on circumscribed choroidal hemangioma has been investigated. The results from this study demonstrated the effectiveness of the suggested therapy in reduction of tumor diameter and thickness of the 18 treated patients. This could be explained by ICG molecules that convert energy into heat to damage vascular endothelial cells after laser irradiation, which leads to the accumulation of endothelial cells so as to form thrombosis, thus blocking blood vessels and contributing to the achievement of therapeutic goals [155]. It is worth mentioning that while it is acknowledged that light plays a role in our circadian rhythms and concomitantly our health, the duration of exposure of each of these light-responsive DDSs tends to be very short and targeted to a specific location of the eye and should not therefore pose a significant risk in disrupting our circadian rhythms [156–159].

The optimization of light-responsive DDS is the subject of ongoing research for specific tissue niches and applications (variables include location, chemistry underpinning the DDSs, molecular weights of the drugs/bioactives, light sources, etc.) to generate highly efficient and cost-effective treatment modalities. Examples of light-responsive DDSs and their stage of development are highlighted in Table 1.

**Table 1:** Examples of light-responsive DDSs and their stage of development

**Irradiation wavelength**	**Therapeutic agent**	**Examples of chemistry**	**Light-responsive moiety**	**Therapeutic use**	**DDS**	**Stage of development**	**Reference(s)**
365 nm	Model drug (e.g., Coomassie blue, fast green and dextran; and lysozyme, bovine serum albumin and myoglobin)	Photo-crosslinking/de-crosslinking	Anthracene		Hyaluronic acid Hydrogel	In vitro testing	[97]
365 nm	Model drug (Cyanine 5)	Photoisomerization	SP		Nanoparticles	Ex vivo testing	[44]
365 nm	Bevacizumab	Photopolymerization	Polycaprolactone dimethacrylate and hydroxyethyl methacrylate monomers and initiator 2,2-dimethoxy-2-phenylacetophenone	CNV	Hydrogel	In vivo testing	[103]
365 nm	Nintedanib	Photocleavage	*o*-NB	CNV	Nanoparticles	In vivo testing	[82]
365 nm and 420 nm	Model drug (rhodamine B)	Photoisomerization	Azobenzene		Cationic vesicles	In vivo testing	[72]
400–500 nm	Bevacizumab	Photothermal	AuNPs embedded in agarose gels	AMD	Hydrogel	Ex vivo testing	[126]
505 nm	Doxorubicin	Photocleavage	Dicyanomethylene derivatives of CM	Retinoblastoma	Nanoparticles	In vivo testing	[86]
635 nm	Aminolevulinic acid	Photosensitization	Aminolevulinic acid	Periocular basal cell carcinoma	Emulsion	Clinical trial	[154]
689 nm	Model drug (Plasmid DNA)	Photosensitization	dendrimer phthalocyanine		Dendrimers/ polyplex	In vivo testing	[121]
689 nm	Verteporfin	Photosensitization	Verteporfin	CNV	Liposomes	Clinical trial	[153, 160]
808 nm	Doxorubicin	Photothermal	Black phosphorus nanosheets	Posterior capsule opacification	IOL	In vivo testing	[137]
810 nm	Indocyanine green	Photothermal	Indocyanine green	Choroidal hemangioma	Solution	Clinical trial	[155]

## Conclusion

A variety of conventional DDSs exist to treat ocular issues; however, problems with such systems exist which motivates the development of stimuli-responsive DDSs as a solution. The literature reviewed highlights the potential of light-responsive DDSs to exhibit a high degree of control for the release of drugs and enhanced ocular bioavailability for a variety of therapeutics. There are ongoing efforts towards clinical translation of stimuli-responsive biomaterials to address problems for ophthalmic applications, which may involve the use of light-responsive biomaterials owing to their versatility.

It is noteworthy that the development, regulation, and adoption of products is a long and difficult process (ca. 15 years to bring a new product to market). There are many points at which a product may be rejected on the grounds of safety, effectiveness, quality, or price over the course of preclinical studies, 3 clinical trial phases, and nation-specific regulation/adoption pathways; consequently, some nations have schemes to offer companies an expedited pathway to market by offering patients with life-threatening or debilitating conditions access to medicines which are not yet market authorized. From an economic point of view, the growing use of stimuli-responsive biomaterials will have economic, health, and societal impacts (potentially alleviating pressure on healthcare systems worldwide, particularly if they can be administered with high patient compliance); we believe that the promising results generated using this exciting class of biomaterials will inspire readers in academia/industry.

## Data Availability

Not Applicable.

## Declarations

### Disclaimer

We note that the views expressed in the review are those of the authors and not necessarily those of the UK National Health Service (NHS), the UK National Institute for Health Research (NIHR), or the UK Department of Health.
